# Point-of-care ultrasound assessment of transrectal diameter to evaluate fecal load in children with anorectal malformations: adjunct imaging and utility in bowel management program

**DOI:** 10.1007/s00383-026-06520-5

**Published:** 2026-07-17

**Authors:** Judith Lindert, Daniel Erkel, Naemi Kurzweil, Joshua Hein, Stefanie Märzheuser

**Affiliations:** Department of Pediatric Surgery, Pediatric Colorectal Centre, University Hospital Rostock, Rostock, Germany

**Keywords:** Bowel management, Anorectal malformation, Point of care ultrasound, Visualisation faecal load

## Abstract

**Introduction:**

Children with anorectal malformations (ARM) frequently experience impaired bowel function and require long-term bowel management. Assessment of fecal load commonly includes abdominal radiographs, which provide information on overall stool distribution but may require repeated imaging and are subject to interobserver variability. Ultrasound measurement of the transrectal diameter (TRD) has been proposed as a radiation-free adjunct in functional constipation, but evidence in children with ARM remains limited. This study aimed to evaluate point-of-care ultrasound (POCUS) measurement of TRD as an adjunct to clinical assessment for evaluating fecal load and monitoring bowel management in children with ARM.

**Methods:**

In this prospective observational cohort study, children with ARM attending a tertiary colorectal clinic between April 2023 and January 2025 underwent standardized ultrasound assessment of TRD as part of a structured bowel management program. Findings were compared with children with functional constipation (FC) and healthy controls. TRD measurements were correlated with clinical symptoms of constipation and soiling. Receiver operating characteristic (ROC) analysis was performed.

**Results:**

Fifty-seven children with ARM (mean age 5.7 years) were included, alongside 53 children with FC and 72 controls. Asymptomatic ARM patients demonstrated TRD values comparable to controls (2.20 vs. 2.05 cm, *p* = 0.246), whereas symptomatic ARM patients showed significantly increased TRD (3.64 cm, *p* < 0.001). ROC analysis identified a TRD cut-off of 3.1 cm with a sensitivity of 63.3% and specificity of 96.3%. During structured bowel management, TRD decreased below this threshold in parallel with clinical improvement.

**Conclusion:**

POCUS measurement of TRD is a feasible, radiation-free adjunct to clinical assessment in children with ARM. While it does not replace radiographic evaluation of global colonic stool burden, it may support monitoring within structured bowel management programs.

## Introduction

Children with anorectal malformations (ARM) are known to suffer from impaired bowel function and frequently require long-term bowel management to achieve social continence [1]. Impaired bowel function adversely affects psychosocial well-being and quality of life, underscoring the importance of structured, individualized management strategies [2, 3].

Bowel management approaches vary depending on age, clinical setting, and family preferences. A range of therapeutic strategies, including laxatives, suppositories, enemas, and transanal or antegrade irrigation, is employed to relieve symptoms and achieve continence [2, 4]. Objective assessment of fecal load is essential for guiding these therapies and monitoring treatment success.

Radiographic imaging remains widely used to assess fecal load, as it provides information on overall stool burden, distribution of fecal load, and colonic dilation [5]. Interpretation remains variable, and repeated imaging may be required during treatment [6, 7]. Several scoring systems have been proposed to quantify fecal loading on abdominal radiographs [7–11], although none has been universally adopted. More recently, structured radiographic protocols have been introduced to standardize interpretation during bowel management programs [12]. Children are repeatedly exposed to radiation. The diagnostic value of abdominal radiographs in constipation remains debated, with limited reliability reported in systematic reviews [12].

Ultrasound has emerged as a radiation-free modality that can be performed as point of care examination at the bedside. Measurement of the transrectal diameter (TRD) has been described in children with functional constipation and shown to correlate with fecal loading [13–17]. A recent systematic review highlighted its diagnostic potential but also emphasized variability in cut-off values and potential age-related influences [18]. Transabdominal ultrasound measurement of rectal diameter has been shown to correlate with fecal loading in pediatric populations and may provide a non-invasive adjunct to clinical assessment [18, 19].

Children with colorectal malformations have largely been excluded from these studies [4, 7, 8]. In patients with ARM, anatomical reconstruction and potential chronic dilation of the neorectum may affect the applicability and interpretation of TRD measurements [20]. Ultrasound is reported as an adjunct imaging tool for determining the appropriate irrigation volume in bowel management for children with colorectal pathology [21, 22].

This study aims to evaluate the transabdominal ultrasound measurement of TRD during routine outpatient visits, performed by clinicians themselves as an adjunct to clinical assessment in children with ARM, and to investigate its role in monitoring bowel management within a structured clinical program.

## Methods

This prospective observational cohort study included children with anorectal malformations (ARM) presenting to our tertiary pediatric colorectal clinic for routine follow-up between April 2023 and January 2025. The study was conducted as part of the ReKiSo study (Rectal Diameter in Children with Colorectal Conditions), a prospective institutional initiative evaluating ultrasound-based assessment of fecal load in pediatric colorectal disorders [4, 7].

**Fig. 1:**
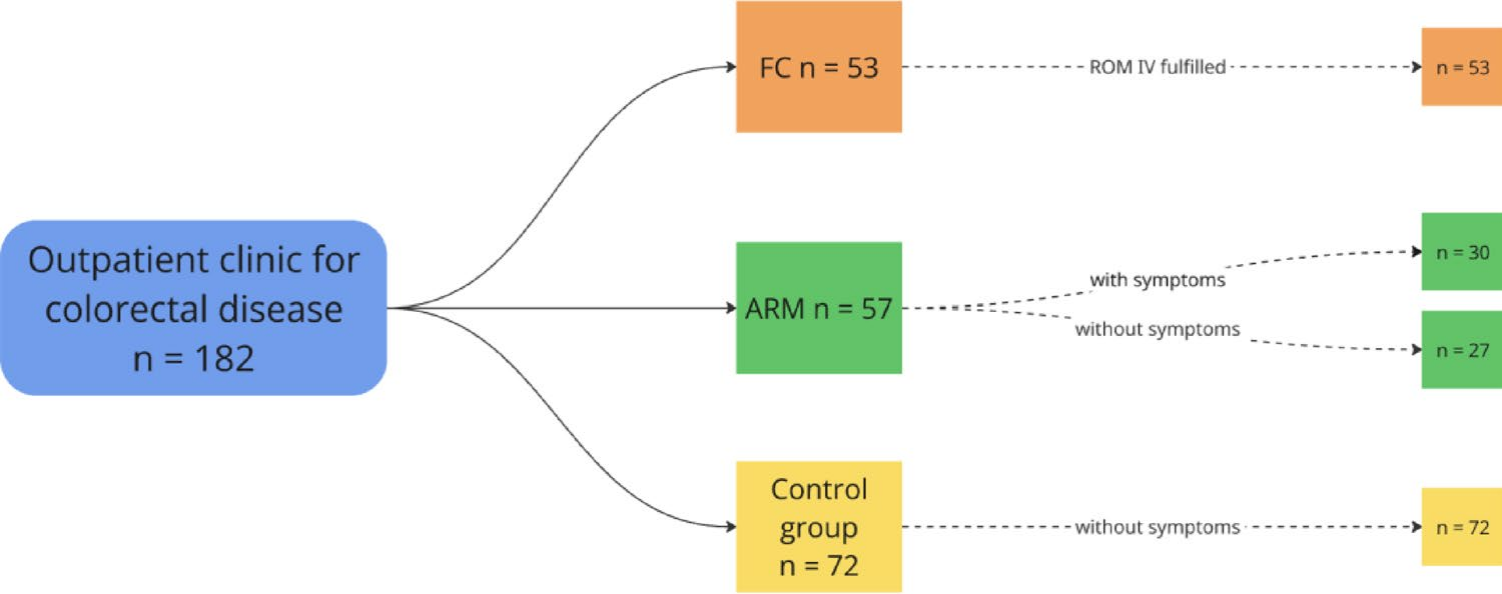
Patient cohort flowchart

Patients were eligible for inclusion if they had undergone surgical reconstruction for ARM and were attending long-term follow-up. Both patients treated at our institution and those referred from external centers were included. In this analysis, only Children with ARM, healthy controls, or functional Constipation have been included, see Fig. 1. Exclusion criteria comprised children with Hirschsprung disease, neurogenic bowel dysfunction (such as spina bifida), and patients younger than one year or older than 18 years.

Clinical assessment was performed using a standardized symptom checklist, which included constipation, stool smearing, painful defecation, large-volume stools, palpable stool retention, and abdominal distension. In children with functional constipation, the Rome IV criteria were applied, and stool consistency was classified according to the Bristol Stool Chart [21].

Clinical decision-making regarding bowel management, including the initiation or modification of laxatives, enemas, or irrigation, was primarily based on clinical symptoms, patient history, and physical examination in accordance with established bowel management principles [2, 4]. Ultrasound findings were used as an adjunct to support clinical assessment and to monitor response to therapy. Abdominal radiographs were not routinely performed in this study and were therefore not used as a reference standard.

**Fig. 2:**
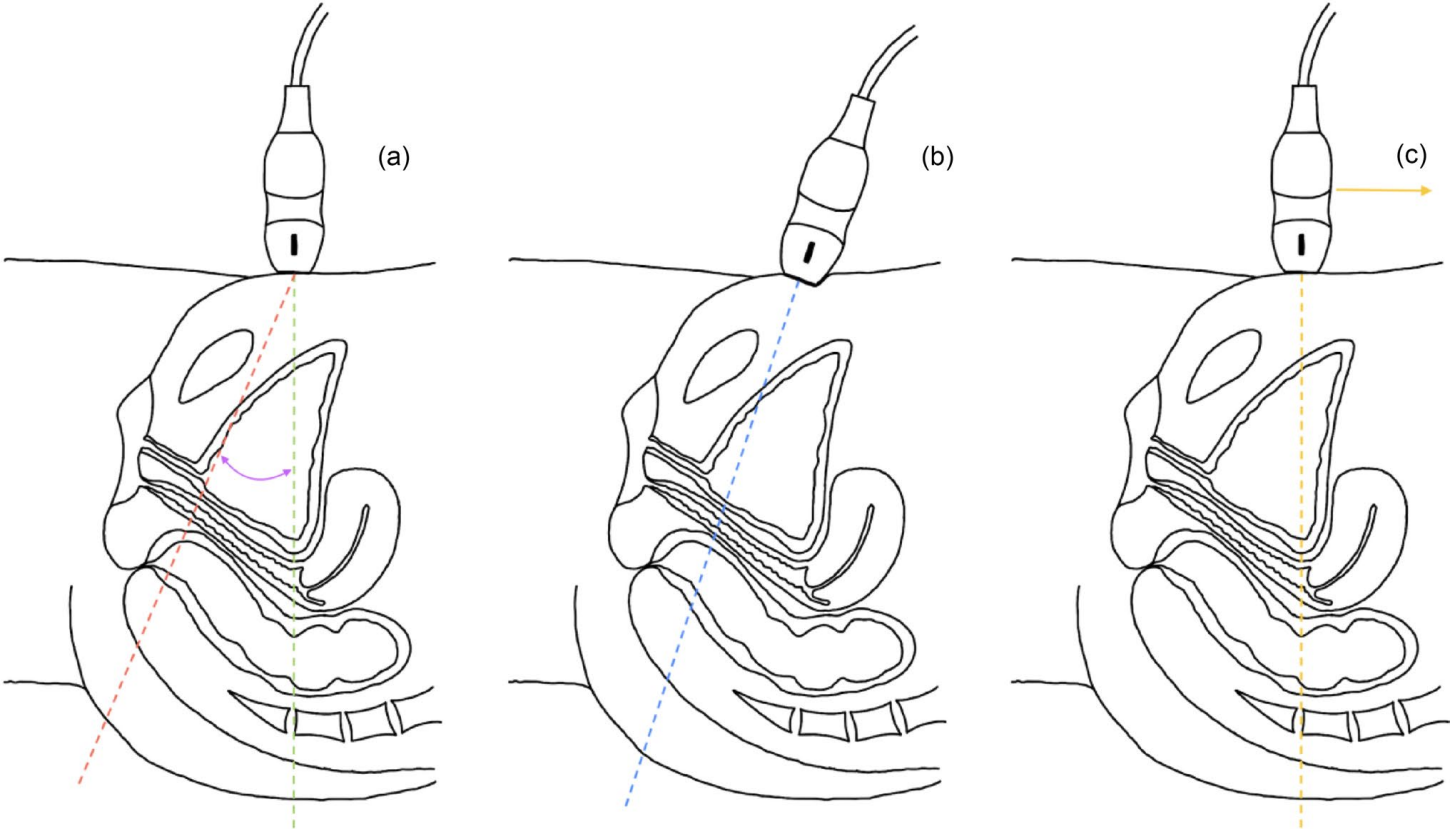
Schematic illustration based on standard anatomical references. **a** overview of sigma and rectum, **b** measurement of TRD, **c ** evaluation of fecal load (© Lindert & Erkel)

Point-of-care ultrasound examinations were performed by a pediatric surgeon and a supervised medical research student using previously described techniques [4, 7, 19]. Patients were examined in the supine position; in younger or less cooperative children, a semi-seated position on a caregiver’s lap was used to improve compliance. A curved array transducer (3.5 MHz) was positioned suprapubically with a slight caudal angulation, see Fig. 2. The examination included visualization of the sigmoid colon, measurement of the maximal horizontal transrectal diameter (including the rectal wall), and qualitative assessment of fecal load in a retrograde fashion from the rectum. When possible, a partially filled urinary bladder was used as an acoustic window. All images were stored electronically and documented in the patient record.

Bowel management followed a structured institutional protocol including macrogol-based laxatives and, where indicated, transanal irrigation using commercially available systems [2, 22–25]. Hydrosonography was selectively employed during the initiation of irrigation to estimate appropriate irrigation volumes [22].

Follow-up was usually done 4–6 weeks later, and for patients living far away, follow-up was ensured via telephone, including repeat ultrasound measurement of the transrectal diameter. Further follow-up was individualized based on clinical progress.

For comparative analysis, children with functional constipation and healthy controls without colorectal pathology were included. Statistical analysis was performed using SigmaPlot 13.0. Normal distribution was assessed using the Shapiro–Wilk test. Group comparisons were conducted using t-tests or Mann–Whitney U tests as appropriate. Correlation between TRD and clinical symptoms was assessed using rank biserial correlation. Receiver operating characteristic (ROC) analysis was performed to determine diagnostic performance and optimal cut-off values.

To address the question of user dependency, interrater reliability was calculated for functional constipation and ARM patients when images had been taken by two independent examiners.

Ethical clearance was obtained from the University of Rostock (protocol code A2023-0066, approved on April 6, 2023). Informed consent was obtained from legal guardians and, where appropriate, from the patients themselves. The study adhered to the principles of the Declaration of Helsinki and was conducted in compliance with good clinical practice guidelines.

## Results

A total of 57 children with anorectal malformations (30 females and 27 males) were included in the study. The mean age was 5.7 years (SD 3.3). These patients were compared with 72 healthy controls and 53 children with functional constipation, see Table 1.

**Table 1:** Overview of patient cohorts

	Anorectal malformation *n* = 57	Control group *n* = 72	Functional constipation *n* = 53
Sex (female: male)	30:27	35:37	23:30
Age years (mean, SD)	5.7 (3.3)	6.9 (3.7)	6.6. (3.7)
Height cm (mean, SD)	108.8 (23.9)	124.2 (24.6)	117.4 (21.5)
Weight kg (mean, SD)	19.8 (11.4)	28.3 (14.3)	25.2 (15.2)

**Fig. 3:**
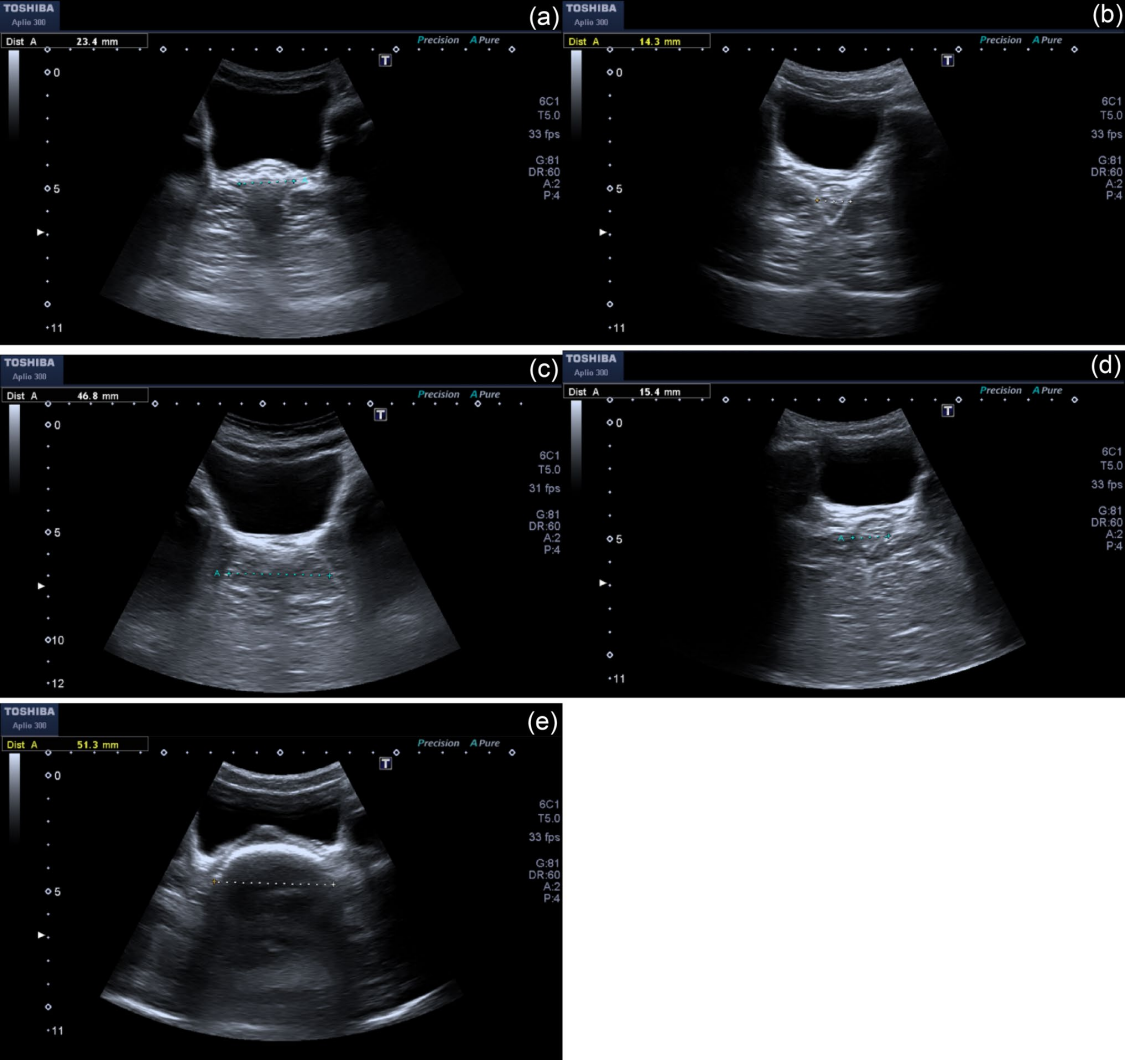
Visualizes the ultrasound measurements of the transabdominal transrectal diameter in **a** a healthy control without symptoms **b** a child with ARM and no symptoms **c** a child with ARM and symptoms, and **d** following successful bowel management, **e** a child with FC and ROM-IV criteria fulfilled

Among children with ARM, asymptomatic patients demonstrated a mean transrectal diameter (TRD) of 2.20 cm, which was not significantly different from that of healthy controls (2.05 cm; *p* = 0.246). In contrast, symptomatic children with ARM showed a significantly increased TRD, with a mean value of 3.64 cm (*p* < 0.001). Figure 3 visualizes the ultrasound appearance of the neorectum and examples of patients.

The correlation between TRD and clinical symptoms in children with ARM was moderate to strong, with a rank biserial correlation coefficient of 0.685, indicating that larger rectal diameters were associated with the presence of symptoms.

Receiver operating characteristic (ROC) analysis identified an optimal TRD cut-off value of 3.1 cm for distinguishing symptomatic from asymptomatic patients. This threshold yielded a sensitivity of 63.3% and a specificity of 96.3%, with a positive likelihood ratio of 17.12.

To address the query of user dependency in doing this point-of-care ultrasound measurement. We could include image pairs for both ARM and FC obtained independently by two examiners. We included 54 image pairs for Anorectal Malformation (29 male, 25 female, mean age 5,37 years) with an ICC of 0.919 (95%CI 0.865–0.952) as well as 102 paired measurements for functional Constipation (58 male, 45 female, mean age 7.35 years) with an ICC of 0.956 (95%CI 0.936–0.970).

**Fig. 4:**
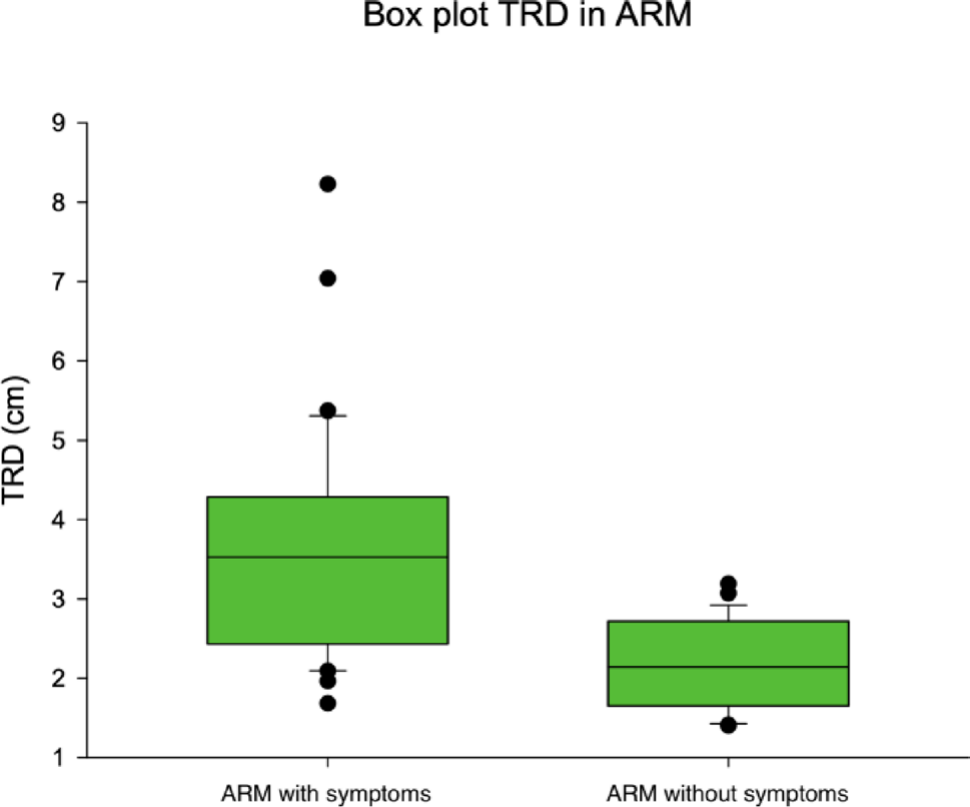
Boxplot mean TRD measurements in children with ARM with and without symptoms

During follow-up under structured bowel management, a reduction in TRD was observed in parallel with clinical improvement. In patients who achieved symptom control, TRD values decreased below the 3.1 cm threshold. The normalization of TRD occurred at a mean follow-up of approximately three months in female patients and six months in male patients, see Fig. 4.

Changes in bowel management were individualized. In some asymptomatic patients, therapy was intensified as part of a proactive strategy to maintain social continence and prevent recurrence of symptoms, consistent with established bowel management principles [2, 26, 27] (see Table 2).

**Table 2:** Transabdominal measurement of the rectal diameter, monitoring bowel management

	ARM without symptoms *n* = 27	ARM with symptoms *n* = 30	Control without Symptoms *n* = 72	Functional Constipation *n* = 53	*p*-value
Change in bowel management initiated	Therapy intensified: 60% therapy unchanged: 40% therapy deescalated: 0%	Therapy unchanged:70%, therapy intensified:19%, therapy deescalated 11%	-	Therapy intensified: 87%, therapy unchanged:11%, therapy deescalated 0%	-/-
Months last follow-up (mean)	6	-	-	3	0.097*
Transabdominal rectal diameter at start (mean, SD)		3.64 (1.47)			-/-
Transabdominal rectal diameter TRD last follow-up (mean, SD)	2.2 (0.56)	2.01 (0.37)	-	2.50 (0.35)	CG vs. ARM_symptomatic_: <0.001* ARM_symptomatic_ vs. ARM_follow-up_: 0.0236** CG vs. ARM_asymptomatic_: 0.246* CG vs. ARM_follow-up_: 0.706* CG vs. FC: **< **0.001*

*Mann-Whitney U test

**One-tailed paired t-test

The rank biserial correlation for the TRD and symptoms of the checklist in ARM was 0.685. This indicates a larger TRD for symptomatic patients with ARM. Based on a positive likelihood ratio of 17.12, the calculated cut-off for the TRD in the ARM cohort was 3.095 with a sensitivity of 63.33% and a specificity of 96,30%.

## Discussion

This prospective study demonstrates that clinician-performed point-of-care ultrasound (POCUS) measurement of the transrectal diameter (TRD) is feasible in children with anorectal malformations (ARM) and correlates with clinical symptoms of fecal loading. Our findings suggest that TRD measurement can serve as a useful adjunct to clinical assessment within structured bowel management programs, particularly for longitudinal monitoring.

A key finding of this study is the description of the sonographic anatomy of the neorectum in children after corrective surgery for anorectal malformation. We can show that the neorectum follows the same sonomorphological pattern as in healthy controls or functional constipation. In children without symptoms regardless of the underlying pathology, the rectum/neorectum has a diameter below 3 cm. We also report the significant difference in TRD between symptomatic and asymptomatic children with ARM. While asymptomatic patients demonstrated rectal diameters comparable to healthy controls, symptomatic patients showed a markedly increased TRD, supporting the concept that rectal distension reflects fecal retention. The observed correlation between TRD and clinical symptoms (rank biserial correlation 0.685) is consistent with previous studies in children with functional constipation, where ultrasound-based rectal diameter measurement has been shown to reflect stool burden [13–18]. Our study extends these findings to a population with surgically reconstructed anorectal anatomy, which has largely been excluded from prior investigations.

However, TRD measurement must be interpreted within its limitations. Unlike abdominal radiography, which provides a comprehensive overview of stool distribution throughout the colon, TRD represents a localized measurement of the rectum. Radiographs allow assessment of global stool burden, segmental distribution, and colonic dilation patterns, which remain important in clinical decision-making [5, 6]. This one point-of-care measurement seems to reflect the overall status of the bowel management quite well and can serve as a point-of-care test. Furthermore, the diagnostic utility of abdominal radiographs has been debated, with systematic reviews demonstrating variable reliability and interobserver agreement [12]. In this context, ultrasound should be seen as a complementary tool that may reduce the need for repeated imaging in selected clinical scenarios and allow the reduction of exposure to radiation.

Before this study, it was not clear if the neorectum may be anatomically altered after corrective surgery. We see an adaptation of the neorectum to the characteristics of the native rectum. Chronic rectal distension may alter urological and anorectal function, including sensation and motility, influenced by the relationship between rectal size and adjacent organs [20]. This highlights the importance of also interpreting TRD measurements longitudinally. In our cohort, normalization of TRD during bowel management was associated with clinical improvement, supporting its role as a monitoring parameter.

In our study, therapeutic adjustments were primarily guided by clinical symptoms and the obtained measurement of the transabdominal transrectal diameter, in line with established bowel management principles [2, 4]. Ultrasound findings were used to support these decisions and to provide an objective parameter for follow-up. This reflects a proactive longitudinal follow-up and management strategy aimed at achieving social continence and preventing fecal retention, rather than treating acute symptoms alone [2, 26, 27]. In this context, TRD measurement may contribute to early identification of subclinical stool retention and facilitate timely intervention. Particularly in adolescence or other patients, where history may differ from the actual reality, we find this supporting bedside imaging useful.

The integration of POCUS into clinical workflows is another important consideration. While ultrasound offers advantages as a radiation-free, bedside modality, its implementation depends on the availability of trained clinicians and appropriate infrastructure. In high-volume pediatric colorectal centers, clinician-performed ultrasound can be readily incorporated into outpatient assessments and may enhance both clinical evaluation and patient education. Visualization of rectal distension can help families better understand the underlying pathophysiology and improve adherence to bowel management regimens. However, in settings with limited access to ultrasound expertise, routine implementation may be more challenging. Therefore, the applicability of this approach may vary across institutions.

Our study also highlights the potential role of ultrasound within structured bowel management programs. Such programs are well established as the cornerstone of achieving social continence in children with ARM and other colorectal conditions [2, 19, 22, 27, 28]. Monitoring treatment response is essential, and while radiographic imaging has traditionally been used for this purpose, ultrasound offers a non-invasive alternative for repeated assessments. In our cohort, TRD decreased below the defined threshold of 3.1 cm in parallel with clinical improvement, suggesting that ultrasound may be useful for tracking treatment response over time. We also could show that the interrater variability for this point-of-care test is good, despite ultrasound often being judged to be user-dependent.

Nevertheless, several limitations must be acknowledged. First, the study did not include a radiographic comparator, as in our settings, we would not do X-rays to diagnose fecal retention following current national guidelines [28–30], and therefore does not allow direct evaluation of the relative diagnostic performance of ultrasound versus abdominal radiography. Second, TRD represents a localized measurement and may not capture proximal stool burden or segmental colonic dilation. Third, anatomical variability in ARM patients may influence baseline rectal diameter and limit the generalizability of cut-off values. Fourth, the sensitivity of TRD for detecting fecal loading was moderate, indicating that it should not be used as a standalone diagnostic tool.

Future studies should aim to validate these findings in larger, multicenter cohorts and explore the relationship between TRD, radiographic findings, and functional outcomes. The integration of ultrasound into standardized bowel management protocols, including its role in guiding therapeutic adjustments, warrants further investigation.

## Data Availability

No datasets were generated or analysed during the current study.
